# Contributory factors to early neonatal deaths in the Upper East Regional Hospital in Ghana

**DOI:** 10.4314/gmj.v57i2.7

**Published:** 2023-06

**Authors:** Francis K. Wuni, Margaret W. Kukeba, Yakubu Zakariah, Emmanuella A. Nyaabila, Aiden S. Saanwie

**Affiliations:** 1 Upper East Regional Hospital, Bolgatanga, Ghana; 2 CK Tedam University for Technology and Applied Sciences, Navrongo, Upper East Region, Ghana

**Keywords:** Death, Ghana, Newborn, Perinatal death

## Abstract

**Objective:**

This study examined factors identified during early neonatal death audits contributing to preventable newborn deaths at the Upper East Regional Hospital.

**Method:**

Data for this study was collected retrospectively from perinatal death audit forms using three data collectors. Data collection lasted two weeks, from 18^th^ June to 2^nd^ July 2021. The data collectors submitted 113 filled hard copy data collection forms. This was then entered into a designed Excel sheet and exported to STATA software version 15.0 for analysis. The analysis was descriptive statistics with cross-tabulation. The results were presented in charts and tables focusing on percentages.

**Results:**

Most of the 113 neonatal deaths were from birth asphyxia (63%). Forty-six (40.7%) of the deaths occurred within 24 hrs after birth. There were 38 factors reported 254 times in the audits as contributory to all the newborn deaths; 17 health personnel-related factors stated 141 (55.5%) times, four transportation and communication-related factors stated 43 (16.9%) times, seven health facility factors stated 31 (12.2%) times. Inappropriate care during transportation to the regional hospital was reported most - 21 times, followed by delay in referral - 18 times.

**Conclusion:**

The study identified many factors, such as medical personnel-related factors, transportation and communication factors, family-related factors, and health facility administration factors, contributing to early neonatal deaths. Effective implementation of neonatal death audit-based recommendations arising from these contributory factors is critical to preventing avoidable newborn deaths.

**Funding:**

This project was funded by Upper East Regional Hospital

## Introduction

Globally, an estimated 5.3 million children under five died in 2018; newborn deaths accounted for 2.5 million.^1^ Most newborn deaths (within the first 28 days of life) occur in low and middle-income countries.^2^ In 2018, while the global neonatal mortality rate was 18 deaths per 1,000 live births, that of sub-Saharan Africa was 28 deaths per 1,000 live births. A neonate in Sub-Saharan Africa has ten (10) times the risk of death than his/her counterpart in a high-income country.^1^ Ghana had a neonatal mortality rate of 25 deaths per 1,000 live births in 2017^1^, and that of the Upper East Region was found to be 24 deaths per 1,000 live births.^3^ Early neonatal death occurs within the first seven days of life.^3^ It accounts for 65 – 80% of all neonatal deaths^3^,^4^ and often presents much stress to the family. Available evidence suggests gaps during delivery in many health facilities. A study assessing the quality of care during delivery in India found that some deliveries were not monitored during labour.^5^ Most health facilities in Africa do not have the required delivery equipment.^6^ Transportation and socioeconomic issues during childbirth have affected many deliveries in Africa.^7^,^8^ The causes and contributory factors of perinatal deaths are mostly preventable in Africa.^9^,^10^ However, it is important to understand the factors contributing to neonatal deaths. One way of identifying the causes and contributory factors of such deaths is a perinatal audit.^11^ A study conducted in Uganda found that perinatal death audit is associated with reduced newborn deaths,^12^ though the latter depends on the effective implementation of recommended interventions.^13^ Ghana, through her Health Services, has developed a national perinatal death audit tool and guidelines to enable facility base assessment and evaluation of these gaps' impact on neonatal deaths.^14^ By 2016, staff were trained on the use of the guidelines.

Despite implementing early neonatal death audits in the country, there is no systematically gathered evidence on the contributory factors as outlined by the national guidelines. In this way, healthcare institutions cannot see the trends in the contributory factors to implement recommended solutions to deal with these factors that occur repeatedly. The uniqueness of this study is that it is the first local study to concentrate on audit-identified contributing factors for only early-neonatal deaths. Although earlier perinatal studies looked at contributing factors, they did so regarding maternal deaths.^15^,^16^

The study objectives were to examine neonatal demographics of newborn deaths that occur within seven days of life at the Upper East Regional Hospital, and to examine factors identified during early neonatal death audits that contributed to preventable newborn deaths.

## Methods

### Study setting

This study occurred at the Upper East Regional Hospital. The facility's newborn care unit was established on 29^th^ January 2014. It has four main sub-units. These include the high dependency, stable/ low dependency, homeward and Kangaroo Mother Care (KMC) room with five beds for continuous KMC practice. An early neonatal death audit started in the hospital in 2017. From January 2019 to December 2020, the unit had 156 early neonatal deaths. Eighty-seven (87) deaths were recorded in 2019, while the remaining sixty-nine (69) deaths were in 2020. Within the same period, the Unit had organised fifty-three (53) death audits for one hundred and twenty-nine (129) early neonatal deaths. Forty-nine of the audits took place in the Regional Hospital, whiles four took place at some of its referring facilities, namely Walewale District hospital, Kalbeo CHPS Compound, Dachio Health Centre and Matyrs of Uganda Health Centre at Sirigu. All the study participants, some referred from these facilities, died at the Regional Hospital.

### Study population, design and sample size determination

All early neonatal deaths at the Newborn Care Unit of the Upper East Regional Hospital from January 2019 to December 2020. However, the target population was audited for early neonatal deaths with a gestational age of 28 weeks or above within seven days of life.

A retrospective cross-sectional study collected data from audited cases from January 2019 to December 2020. The facility's institutional neonatal mortality has been 8% yearly. We used Cochran's sample size formula N= (z)^2^ p (1-p) ÷m^2^ to estimate the sample size for the study^15^. Where N= sample size required, p=prevalence of institutional neonatal mortality at 8% and m=margin of error at 5% (0.05). Early neonatal deaths accounts for 65 – 80% of all neonatal deaths^3^,^4^ yearly. Considering 65% of 113 neonatal deaths gave us 74 early neonatal deaths in a year. For the 2 years, the sample size was expected to be 148. However, only one hundred and twenty-three (123) audit forms were retrieved for data collection for the 2 years. Ten forms were excluded, three of which had a diagnosis of congenital anomaly, while the remaining seven had incomplete documentation. The remaining 113 neonatal deaths met the inclusion criteria and were included in the study.

### Data collection

Three nurses were recruited for data collection. They were taken through the study purpose, objectives and data collection procedures. The data for this study were collected retrospectively from the perinatal death audit forms for audited newborn deaths. Each neonate's perinatal death audit form included information about the mother and neonate demographics, the cause of death, and contributory factors discovered during the audit. The forms were obtained from the Upper East Regional Hospital. The investigators designed a data collection tool for the data collection. It was pre-tested on 15 death audit forms on 3^rd^ June, 2021 to ensure its suitability. Data collection started on 18^th^ June, to 2^nd^ July, 2021.

### Data Analysis

The data collectors submitted 113 filled hard copy data collection forms. These were then entered into a designed Excel spreadsheet by the investigators and exported to STATA software version 15.0. The investigators cleaned the data for language errors before data analysis. The data was then analysed using STATA. The analysis was descriptive statistics with cross-tabulation.

### Ethical Consideration

The study was approved by Navrongo Health Research Center Institutional Review Board with ID number NHRCIRB392. The Upper East Regional Director of Health Services granted permission to conduct the study via a letter. No contributory factor identified in the study is linked with a particular health facility. This was done to ensure confidentiality. The data was secured against unauthorised access. Every person who handled the data complied with our privacy policy.

## Results

### Demographics

The study analysed one hundred and thirteen (113) audited forms. Sixty-three of them were males (55.7%). Most of the participants 85/113 (75.2%) were delivered by spontaneous vaginal delivery.

Forty-six (40.7%) of the participants died within 24hrs after birth. Death within 24hrs of life was higher among participants delivered through cesarean section, 67.9% (19/28). Fifty-one (45.1%) of the participants were delivered at the Regional Hospital. Of the 113 neonates, 24.8% were born to maternal age 25-29. Table 1 shows the detailed demographic characteristics of the study participants.

**Table 1:** Demographic characteristics of participants

Demographic characteristic	n(%)
**Sex**	
Male	63(55.7)
Female	50(44.3)
**Mode of delivery**	
Spontaneous vaginal Delivery (SVD)	85(75.2)
Cesarean Section	28(24.8)
**Day(s) of life**	
Died within 24hrs of life	46(40.7)
Died between 1-7days of life	67(59.3)
**Place of delivery**	
Home delivery	2(1.8)
CHPS Compound	13(11.5)
Health Centre	18(15.9)
Private Hospital	14(12.4)
District Hospital	15(13.3)
Regional Hospital	51(45.1)
**Maternal Age**	
**15-19**	27(23.9)
**20-24**	27(23.9)
**25-29**	28(24.8)
**30-34**	18(15.9)
**35-39**	10(8.9)
40+	3(2.6)

### Causes of early neonatal deaths

There were five causes of death identified in the study. Most deaths were caused by birth asphyxia (71, 63%). Table 2 shows the causes of death.

**Table 2:** Causes of early neonatal deaths at the Upper East Regional Hospital, Ghana (n=113)

Diagnosis	n(%)
**Birth asphyxia**	71(63)
**Prematurity**	31(27)
**Neonatal sepsis**	7(6)
**Neonatal jaundice**	3(3)
**Anaemia**	1(1)

### Contributory factors to deaths

Overall, there were 38 factors reported 254 times in the audits. Of these factors, there were 17 factors related to health personnel and these came up 141 times, 4 transportation and communication-related factors reported 43 times, 10 *mother* and family-related factors reported 39 times and 7 health facility administrative factors reported 31 times.

### Medical personnel related contributory factors

The skills, knowledge and attitude of staff in the perinatal care environment greatly contributed to these deaths, as shown in table 3. Of 17 factors related to these skills, attitude and knowledge of health workers in the perinatal care setting in this study, delay in referring patient for secondary care is the most contributory factor (12.8%). This was followed by management of 2^nd^ stage labour prolonged with inappropriate interventions, and inadequate neonatal care management by health staff (10.6% each). Staff inability to diagnosed breech presentation until late labour contributed the least (1.4%).

**Table 3:** Medical personnel-related factors to early neonatal deaths at the Upper East Regional Hospital, Ghana (n=141)

Contributory factors	n (%)
Delay in referring patient for secondary care	18(12.8)
Management of the second stage prolonged with inappropriate intervention	15(10.6)
Neonatal care: management plan inadequate	15(10.6)
Neonatal care: inadequate monitoring	14(9.9)
Neonatal resuscitation inadequate	11(7.8)
Delay in medical personnel calling for expert assistance	10(7.1)
Foetal distress not detected during ANC or intrapartum	7(5.0)
Inappropriate response to apparent post-term pregnancy	7(5.0)
Poor estimation of foetal size by medical personnel	7(5.0)
Baby managed incorrectly at Hospital/Clinic	6(4.3)
Incorrect management of complications	6(4.3)
Inappropriate management of complications when they set in during antepartum period	5(3.5)
Incorrect diagnosis	5(3.5)
Management of the second stage prolonged with no intervention	5(3.5)
Baby sent home inappropriately when not well	4(2.8)
Partogram used to monitor labour, but not interpreted correctly	4(2.8)
Breech presentation not diagnosed until late labour	2(1.4)

### Transportation and communication factors

Table 4 shows that transportation factors contributed to the deaths of some of the study participants. Out of the four factors in Table 3, inappropriate care during transportation to receiving facility accounted for 48.8%.

**Table 4:** Transport and communication factors in early neonatal deaths at the Upper East Regional Hospital, Ghana (n-43)

Contributory factors	n(%)
Inappropriate care during transport	21(48.84)
Lack of transport between facilities	12(27.91)
Patient transfer not communicated to receiving facility	9(20.93)
Lack of transport - Home to facility	1(2.32)

### Mother and family-related factors

Table 5 showed nine contributory factors to early neonatal deaths linked to the mother and family's knowledge, attitude and health-seeking behaviours. Of the nine factors, mother delay in seeking medical attention during labour contributed to 28.2%. This was followed by delay in seeking care when labour complications set in (20.5%) and booking late in pregnancy (17.9%).

**Table 5:** Mother and family-related factors in early neonatal deaths at the Upper East Regional Hospital, Ghana

Contributory factors	n(%)
Delay in seeking medical attention during labour	11(28.2)
Delay in seeking care when complications set in	8(20.5)
Booked late in pregnancy	7(17.9)
Inadequate visits to antenatal clinic (< 4 visits by 36/52	4(10.2)
No Knowledge of danger symptoms	3(7.7)
Use of herbal medicine	2(5.1)
Never initiated antenatal	1(2.6)
Failed to return on the prescribed date	1(2.6)
Financial Constraints	1(2.6)
Delay in seeking help when baby was ill	1(2.6)

### Healthcare facility administrative factors

As shown in Table 6, health facilities issues such as inadequate staffing at delivery and newborn care settings, inadequate neonatal resuscitation equipment and theatre facilities greatly contribute to the outcome of newborn care. Antenatal care routine laboratory tests not done accounted for 35.5% of all the health facility administrative issues, followed by insufficient nurses on duty to adequately manage the patient (29.0%).

**Table 6:** Healthcare facility administrative factors related to early neonatal deaths at the Upper East Hospital, Ghana (n=31)

CONTRIBUTORY FACTORS	n (%)
ANC routine labs not done (Hb, Syphilis, Blood Group etc)	11(35.5)
Insufficient nurses on duty to manage the patient adequately	9(29.0)
Inadequate facilities/resuscitation equipment in neonatal unit/Nursery)	3(9.7)
Personnel insufficiently trained or too junior to manage the patient	3(9.7)
Insufficient doctors to manage the patient	3(9.7)
Inadequate theatre facilities	1(3.2)
Insufficient blood/ blood products available	1(3.2)

## Discussion

A high early neonatal death rate indicates a nation's healthcare system breakdown. Knowing the circumstances that may have led to these deaths is important. Strengthening perinatal care to prevent similar avoidable deaths can easily be achieved by eliminating circumstances that repeatedly show their ugly heads to these neonates. The study found that many modifiable factors contributed to early neonatal deaths.

In this study, 63% of newborns died from birth asphyxia. A previous study on the causes of newborn deaths in the Ashanti region reported 37.4% of babies dying from birth asphyxia.^9^ Another study in northern Ghana also found that 21% of newborn babies die from asphyxia.^3^ The current study investigated newborn babies who died within 0-7 days of life, whiles the previous studies looked at newborn deaths from 0-28 days of life. This could account for the relatively higher percentage of deaths due to birth asphyxia in this study.

More than half of the contributory factors were medical personnel-related (55.5%) when they were categorised into patient-oriented, medical personnel-related, transportation and communication, and administrative/system-related factors. This finding is consistent with a prior study that identified poor practices by health personnel as the primary contributing factor to newborn deaths in low-resource settings.^17^ These factors relating to medical staff include problems arising from the abilities, expertise, and attitudes of healthcare professionals. In order to improve newborn care outcomes, the skills, knowledge, and attitudes of these professionals working in the perinatal care setting must be improved.

Among the specific medical personnel-related factors, delay in health personnel referring patients for secondary care stands tall (12.8%). This finding is higher than a previous study in Nigeria that reported a delay in referrals at 11.8%.^16^ The study discovered that inadequate neonatal care also contributed significantly to newborn deaths. The organization of newborn care in healthcare facilities is key to adequate management of sick newborns. As a result, stakeholders within the perinatal care environmental are advocating for the creating of newborn care units or corners at every healthcare facility. However, some healthcare-centres that referred some of these neonates to the regional hospital do not have the designated newborn care corners with the needed resources to start with the initial management.

The availability of protocols to guide staff on the care common problems of newborns is also a big issue in some of these facilities. Therefore, there is the urgent need to adequately address these issues to improve the quality of newborn care rendered in these facilities. Future research on the resources and quality of perinatal care rendered at the Health Centres and Community-based Health Planning and Services (CHPS) is also recommended.

We also found inappropriate care when transporting sick neonates for secondary care as a major contributor to newborn deaths. Most of the deaths in this current study were due to birth asphyxia.

It suggests these babies could not initiate or sustain breathing at birth, requiring frequent monitoring and resuscitative services such as oxygen administration or bag and mask ventilation. Healthcare workers stationed in the delivery environment have received much training on neonatal resuscitation in the region. It is still unclear why healthcare personnel cannot provide these needed services when transporting cases for secondary care. A previous study in Tanzania linked inappropriate interventions during childbirth to poor monitoring.^18^ Consistent with previous studies,^7^,^19^,^8^ the findings from this current study showed that lack of transport between health facilities during delivery negatively affects newborn care outcomes. In the entire Upper East Region, access to a gynaecologist is limited to the regional hospital. Complicated deliveries have to be transported to the regional hospital. As shown in this study, the means of transport to often convey such cases on time for secondary care negatively impacts perinatal health care.

Under the patient-oriented factors, our study also revealed that delays in seeking medical attention during labour and when complication arise both have negative effect on neonatal care outcomes. Many factors could contribute to these delays. For instance, a previous study in Ghana discovered a favourable correlation between health staff influences and maternal choices regarding location of birth. The quality of births at medical facilities improves when healthcare professionals have some influence over women's choices about where to give birth.^8^ Therefore, one could conclude that women in labour would most likely seek medical attention early if healthcare workers were able to influence this during antenatal care services. Further research on why such delays by women in labour are recommended. It is also important to evaluate the quality of the perinatal death audit at the regional hospital from which these contributory factors were made.

This study was conducted only on newborn deaths at the Upper East Regional Hospital. As a result, the trend of contributory factors to early neonatal deaths will be limited to this referral Hospital and Hospitals that refer sick neonates to the latter. Additionally, even though the neonatal death audit commenced in the hospital in 2017, the neonatal death audit documents for 2017 to 2018 were reportedly inaccessible to researchers. Thus, the data for this study was only from 2019 to 2020. This tends to limit the generalisation of the findings of this study as the period under review was short.

## Conclusion

The study has identified many factors, such as medical personnel-related factors, transportation and communication factors, mother and family-related factors, and health facility administration factors contributing to early neonatal deaths. The findings also revealed that most deaths were cases of birth asphyxia, and most died within 24 hours of birth. Since most of these contributory factors repeatedly show up, implementing recommendations based on perinatal mortality audits is essential to preventing needless newborn deaths.
